# GS Hip Nail versus Affixus Hip Fracture Nail for the Intramedullary Nailing of Intertrochanteric Fractures

**DOI:** 10.3390/jcm12216720

**Published:** 2023-10-24

**Authors:** Seungcheol Kwon, Minjae Lee, Heeyeon Lee, Jihyo Hwang

**Affiliations:** 1 Department of Orthopedic Surgery, Kangnam Sacred Heart Hospital, College of Medicine, Hallym University, Seoul 07441, Republic of Korea; youthinl@naver.com (S.K.); mjlee1224@hallym.or.kr (M.L.); 2 Department of Engineering of Regenerative, Dongguk University, Seoul 04620, Republic of Korea; hyeon9613@naver.com

**Keywords:** GS hip nail, Affixus hip fracture nail, intertrochanteric fracture, femoral nail

## Abstract

Background: Intertrochanteric fractures are a global health concern, especially in aging populations like the Republic of Korea. Surgical treatments like intramedullary nailing are preferred for their benefit. Various hip nails are used worldwide, each with unique features and challenges. This study aims to compare the GS hip nail with the Affixus hip fracture nail for the treatment of intertrochanteric fractures. Material and Methods: This retrospective study, conducted at a single center, included 179 patients who underwent intramedullary nailing for intertrochanteric fractures using the GS hip nail or the Affixus hip fracture nail. Excluding specific cases, 43 patients in the GS group and 46 in the Affixus group met the minimum 6-month follow-up criteria. Result: The GS group exhibited a significantly shorter mean operation time (43.26 min) compared to the Affixus group (51.11 min). Radiographically, both groups showed no significant differences in their reduction quality, tip, and apex distance (TAD), or Cleveland index in the immediate postoperative window. However, the GS group achieved a greater valgus reduction based on the contralateral femoral neck shaft angle (NSA). At 6 months post-operation, there were no significant differences in TAD or advancement and sliding distances. Complication rates were similar between the two groups, with no implant breakages. Clinical outcomes, as measured via mHHS and EQ-5D-5L, showed no significant differences. Despite a slightly higher implant cost, the GS group had a lower total hospital cost than the Affixus group, but this was not statistically significant. Conclusions: This study highlights the efficiency of the GS hip nail in reducing the operation time compared to the Affixus hip fracture nail with comparable radiologic and clinical outcomes. Further research with long-term follow-up and larger patient studies are needed to fully assess its effectiveness in improving patient outcomes in hip fracture treatment.

## 1. Introduction

The annual incidence of hip fractures worldwide is predicted to increase, and this is attributed to the growing elderly population globally. It is known that the increase in the elderly population is set to become more prominent in Asia and Latin America compared to Europe and North America [1]. The Republic of Korea is the fastest-aging country among OECD nations. By 2018, it had already entered an aged society, with the elderly population (aged 65 and over) surpassing 14%. It is expected to transition into a super-aged society within a few years [2]. An intertrochanteric fracture, the second most common fracture in the proximal femur after femoral neck fracture, is expected to impose significant social and economic burdens [3,4,5]. Appropriate treatment for the tertrochanteric fracture of the femur is not only crucial for the patient’s quality of life but also for social factors. The purpose of surgical treatment is to achieve anatomical reduction and stable fixation, allowing early mobilization and walking to reduce complications associated with prolonged bed rest [6]. Dynamic compression screws and intramedullary nailing are commonly used, with intramedullary nailing gaining popularity in recent years due to its minimally invasive nature, reduced blood loss, shorter surgical time, and biomechanically stable fixation, enabling relatively early mobilization [7,8,9].

There have been numerous clinical and biomechanical studies analyzing the outcomes of various hip nails available worldwide, including the Gamma nail (Stryker, Duisburg, Germany), the Proximal Femoral Nail Antirotation (Synthes, Solothurn, Switzerland), and the Affixus hip fracture nail (Zimmer-Biomet, Warsaw, IN, USA). However, these devices have also encountered issues such as cut-out, implant breakage, femoral shaft fracture, and subsequent loss and reduction in clinical practice [10,11,12,13,14]. In the case of the gamma nail, after surgical treatment of intertrochanteric fractures, there is a potential risk of secondary rotation in the head-neck fragment followed by collapse at the fracture site and cut-out. The reported cut-out rates range from 4% to 8% [15,16,17]. Among these, the Affixus hip fracture nail was developed with a unique design of the lag screw and thread, aiming to demonstrate resistance to cut-out. According to Marbrouk et al. [18], the overall complication rate requiring further surgery was 3%. The cut-out rate was 1%. The back-out rate was 1%. Fracture healing was achieved in all patients. No cases of implant breakage and fatigue were observed during the follow-up period. The Affixus hip fracture nail has a proximal diameter of 15.6 mm and features a nail with 10 degrees of proximal anteversion and 4 degrees of lateral bend. It is available in a single short nail length of 180 mm, and the surgeon can choose from distal diameters of 9, 11, 13, or 15 mm. Additionally, surgeons can select a 10.5 mm lag screw tailored to 125 degrees or 130 degrees neck angles. Furthermore, an additional 5 mm anti-rotation screw can be inserted for the rotational stability of the femoral head.

**Figure 1:**
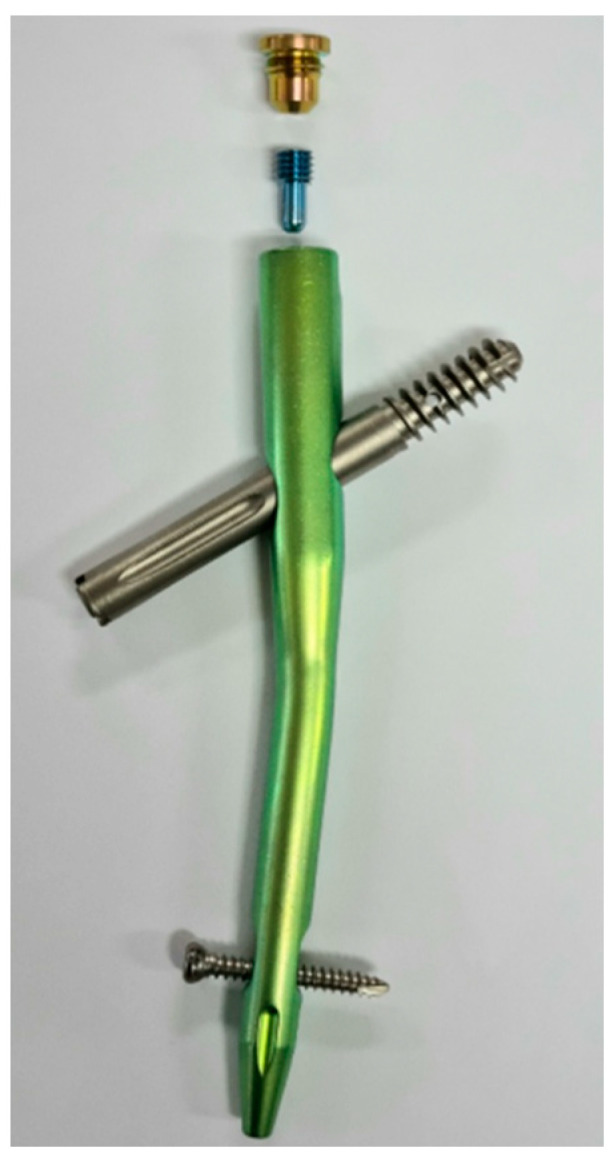
Photograph of the GS hip nail. This is a depiction of the nail, lag screw, and distal screw, along with the set screw that connects the nail and lag screw and the end cap of the nail.

In the Republic of Korea, the GS hip nail (GS medical, Cheongwon-gun, Republic of Korea) has also been developed for the treatment of proximal femoral fractures. Lag screws come in the following two forms: screw-shaped and blade-shaped. The screw-shaped design allows for easy insertion and simple length adjustment, making it easier to achieve fixation in firm bone with the tip located just beneath the articular surface. However, it can lead to fixation failure due to the rotation of the femoral head. On the other hand, the blade-shaped design requires significant force to counteract femoral head rotation, thus preventing varus deformity. However, it has the disadvantage of having a sharp blade that can potentially lead to a cut through [19]. The GS hip nail provides the advantage of a dual system, offering a choice between a blade type and a screw type for the proximal locking mechanism. This allows surgeons to select the appropriate option based on factors such as the patient’s osteoporosis condition, bone quality, and the type of fracture. Both types, once inserted, are securely fixed to the nail body through an internal locking system, effectively preventing rotation movement and reducing the occurrence of cut-out. The GS hip nail has a proximal diameter of 16 mm and features a 5-degree lateral bend. It offers a choice of nail lengths from 170, 180, to 200 mm, and diameters of 10, 11, or 12 mm, making it tailored to the Asian anatomy. The lag screw also has a 10.5 mm diameter and can be inserted at both 125 degrees and 130 degrees with a screw thread length of 32 mm. Considering the shorter length of the femoral neck, the shortest lag screw length can be chosen from 70 mm with 5 mm increments. For the helical blade type, cement can also be injected after insertion (Figure 1).

The primary objective of this study is to compare a reduction in the risk of postoperative complications, such as fracture fixation failure, cut-out, and back-out, when treating intertrochanteric fracture patients using the lag screw type of the GS hip nail, known for its advantages, with those treated using the Affixus hip fracture nail.

## 2. Material and Methods

### 2.1. Study Design and Patients

**Figure 2:**
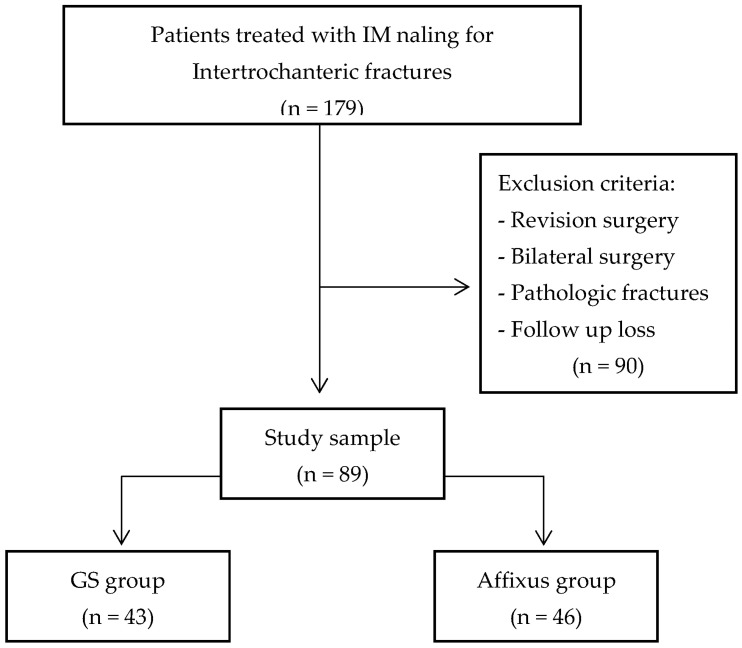
The flow chart of the study population.

This study was conducted after receiving the approval of the Hallym University Kangnam Sacred Heart Hospital Institutional Review Board (IRB) (2023-08-019). This study is a retrospective study conducted at a single university hospital (Kangnam Sacred Heart Hospital, Hallym University, Seoul, Republic of Korea) on a total of 179 patients who underwent surgical treatment for intertrochanteric fractures with the GS hip nail and the Affixus hip fracture nail devices via intramedullary nailing from September 2019 to February 2023. Patients who underwent revision surgery due to the failure of a previous operation, those with pathologic fractures, individuals who underwent bilateral surgery, and patients who were lost to follow-up due to death or other reasons were excluded from the study. Ultimately, 43 patients in the GS hip nail group (GS group) and 46 patients in the Affixus fracture nail group (Affixus group) met the minimum 6-month follow-up requirements (Figure 2).

The demographic data of patients included age, gender, body mass index (BMI), bone mineral density (BMD), time to operation, length of hospital stay, preoperative hemoglobin levels, smoking status, American Society of Anesthesiologists (ASA) score, Koval classification, and AO/OTA classification.

Patients were encouraged to use wheelchairs and initiate training to walk as soon as their general condition permitted. Depending on each patient’s general function and preference, dual crutches or a walker were allowed to support permissible weight-bearing.

### 2.2. Surgical Technique

All surgeries were performed by a single surgeon, with patients placed in a supine position on a radiolucent fracture table under either general anesthesia or spinal anesthesia. Closed reduction was conducted using the C-arm device present in the operating room. After the traction of the fractured limb, a reduction was achieved by internal or external rotation and adduction or abduction, depending on the fracture pattern, followed by a confirmation of the reduction status with an image amplification device. In cases where anatomical reduction was not satisfactory for the anteroposterior (AP) and lateral view, percutaneous techniques were employed using long Kelly-–Rankin hemostatic forceps to assist in fragment reduction. In the AP view, efforts were made to achieve maximal anatomical alignment or a slight valgus alignment with positive medial apposition. In the lateral radiographic view (lateral view), efforts were made to ensure a smooth anterior cortex. After achieving reduction, a guide pin was inserted just medial to the greater trochanter as an entry point. Following this, a reamer was used to create a canal in the proximal femur, and a metal sleeve was inserted. The guide pin for the lag screw was inserted to target the inferior at 1/3 of the head in the AP view, which was aimed centrally in the lateral view. Efforts were made to ensure that the length of the lag screw was 25 mm or less from the tip-apex distance (TAD). In all cases, a 125-degree cephalomedullary nail was inserted, taking into account the neck–shaft angle (NSA) of the femur. A set screw was then inserted in the proximal portion of the metal sleeve to enhance the stability of the lag screw fixation.

### 2.3. Evaluation

First, we measured the operation time (minutes) and blood loss through intraoperative evaluation. To assess the patient’s overall tendency to bleed, we measured the difference between the preoperative hemoglobin levels and the hemoglobin levels on the first day after surgery (POD 1) (Difference = PostOP Hb − PreOP Hb), which was obtained through preoperative blood tests.

For radiologic evaluation, AP and lateral view imaging was performed immediately post-operation, and reduction quality was assessed according to the classification by Chang et al. [20], categorized as good, acceptable, or poor. A good reduction is needed to satisfy normal or slight valgus alignment with positive medial apposition in the AP view and central axial alignment with smooth anterior cortex contact in the lateral view. In this context, if medial displacement is greater than 4 mm or angulation exceeds 20 degrees, the reduction is considered unsatisfactory. Since the maximum cortical thickness is 4 mm, the reduction should result in the translation of 4 mm or less. If the reduction meets either the AP view or the lateral view criteria, it is considered acceptable. If it fails to meet both criteria, it is classified as a poor reduction [20]. We also measured the tip and apex distance (TAD_postOP_) (mm), which is the distance between the attachment of the lag screw and the cortical bone of the femoral head [21]. Additionally, we established the Cleveland index, dividing the region of the femoral head into 9 zones to denote the position of the lag screw [22]. We measured the contralateral femoral neck–shaft angle (NSA) (degrees) in a preoperative radiographic examination. Additionally, after surgery, we measured the operative side’s femoral NSA (NSA_postOP_) and investigated the difference between these two values (Difference = NSA_postOP_ − Contralateral NSA).

Patients were regularly followed up even after discharge (at postoperative 6 weeks, 3 months, and 6 months), and at each visit, radiographic evaluations were performed. Bone union was confirmed when callus or cortical bone continuity was observed in at least three directions along the anterior, posterior, medial, and lateral cortex following the fracture line. The 6 months TAD (TAD_6m_) and the difference between this value and the immediate postoperative TAD (TAD difference = TAD_6m_ − TAD_postOP_) were assessed. Additionally, the 6 month NSA (NSA_6m_) and the difference between this value and the immediate postoperative NSA (NSA_postOP_) were evaluated (NSA difference = NSA_6m_ − NSA_postOP_).

**Figure 3:**
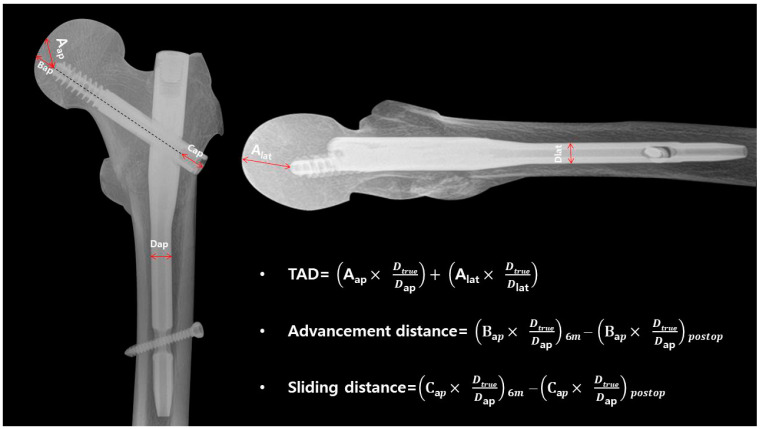
In the AP view of the X-ray for A_ap_, the distance was measured from the lag screw tip to the apex of the femoral head. For B_ap_, the measurement was taken from the lag screw tip along the axis to the point where it intersects with the femoral head. C_ap_ represents the distance from the nail to the lag screw end, and D_ap_ represents the measurement of the nail’s distal diameter. In the lateral view X-ray, A_lat_ represents the distance from the lag screw tip to the apex of the femoral head, while D_lat_ signifies the measurement of the nail’s distal diameter. All measurements were corrected for magnification using the actual length of the diameter.

To assess the fixation strength of the lag screw, we also measured the advancement distance (mm) and sliding distance (mm) [23,24]. For the advancement distance, we measured the distance from the tip of the lag screw along the line drawn from the center axis to where it met the femoral head. The difference between this measurement immediately post-operation and at the 6-month radiographic examination was calculated. Similarly, sliding distance was measured from the point where the lag screw met the nail to the lag screw’s end. The difference in this measurement between immediate postoperative and 6 months during the radiographic examination was also recorded (Figure 3).

All measurements obtained through radiographic examinations, whether in AP or lateral views, were corrected for magnification using the distal diameter of the nail as a reference.

In the final follow-up of radiographic examinations, postoperative complications such as cut-out, cut through, back-out, non-union, delayed union, osteolysis, and implant breakage were investigated. Clinical outcomes were assessed using the modified Harris Hip Score (mHHS) [25,26] at 6 months follow-up, as well as the Euro-Qol-5 Dimension-5 Levels (EQ-5D-5L) [27,28,29] to evaluate patients’ quality of life. Finally, a comparison was made between the implant cost (dollars) and hospital cost (dollars) of the patients.

### 2.4. Statistics

The statistical analysis of these results was conducted using the SAS^®^ 9.4 64-bit program (SAS Institute Inc., Cary, NC, USA). Continuous variables were presented as the mean ± standard deviation. To assess whether data followed a normal distribution, the Shapiro–Wilk test was utilized. If data satisfied the criteria for a normal distribution, a two-sample *t*-test was employed to compare the two groups. If data did not meet the assumptions of normality, Wilcoxon’s rank sum test was applied. Categorical variables were presented as frequencies and percentages. Pearson’s Chi-square test was used to analyze categorical data, and if the expected cell count was less than 5 and exceeded 20%, Fisher’s exact test was utilized. All tests were two-tailed with a significance level of 5%. Decimal places for all values were presented for up to two decimal points. *p*-values were reported for up to four decimal places, and if the *p*-value was less than 0.0001, it was indicated as <0.0001. Statistical significance was considered for *p*-values less than 0.05.

## 3. Results

### 3.1. Demographic Data

In the GS group and Affixus group, the mean ages (years) were 73.02 and 73.20, respectively. Gender distribution in the GS group included 16 males and 27 females, while the Affixus group had 22 males and 24 females. The average BMI (kg/m^2^) was 22.73 for the GS group and 23.97 for the Affixus group, and BMD averaged −3.03 and −2.73, respectively. The time to operation was 3.93 days for the GS group and 2.46 days for the Affixus group, although this difference was not statistically significant. Hospital stay was significantly shorter in the GS group at 13.42 days compared to 17.80 days in the Affixus group. Preoperative hemoglobin levels (g/dL) were similar between these two groups, measuring 12.08 and 12.05, with no statistical difference (Table 1).

In terms of smoking status, the GS group had 6 smokers and 37 non-smokers, while the Affixus group had 4 smokers and 42 non-smokers. ASA scores were distributed as follows: in the GS group, there were 26 patients with an ASA score of 2 and 17 patients with an ASA score of 3, while in the Affixus group, there were 28 patients with an ASA score of 2 and 18 patients with an ASA score of 3. Preoperative Koval grades were as follows: in the GS group, there were 28 patients with grade 1, two patients with grade 2, three patients with grade 3, five patients with grade 4, three patients with grade 5, and two patients with grade 6. In the Affixus group, there were 28 patients with grade 1, 7 patients with grade 2, 1 patient with grade 3, 8 patients with grade 4, and 2 patients with grade 5. Regarding the AO/OTA classification, in the GS group, there were 10 patients with type 11, 8 patients with type 12, 1 patient with type 13, 7 patients with type 21, 10 patients with type 22, 2 patients with type 23, 2 patients with type 31, and 3 patients with type 33. In the Affixus group, there were 8 patients with type 11, 5 patients with type 12, 2 patients with type 13, 10 patients with type 21, 10 patients with type 22, 6 patients with type 23, 2 patients with type 31, and 3 patients with type 33. None of these values showed statistical significance (Table 2).

### 3.2. Intraoperative Evaluation

The mean operation time was statistically and significantly shorter in the GS group, with an average of 43.26 min compared to 51.11 min in the Affixus group. The hemoglobin levels measured on the first day after surgery were 9.26 and 9.16 in the GS and Affixus groups, respectively, with no significant difference between them. Furthermore, the changes in hemoglobin levels compared to preoperative values were −2.82 and −2.89 for the GS and Affixus groups, respectively, and this difference did not reach statistical significance, indicating no significant difference in the extent of hemoglobin decrease (Table 3).

### 3.3. Radiologic Evaluation

In the radiographic assessments conducted immediately post-operation, the GS group had 17 cases of good reduction, 14 cases of acceptable reduction, and 12 cases of poor reduction. In the Affixus group, there were 19 cases of good reduction, 21 cases of acceptable reduction, and 6 cases of poor reduction. These figures did not exhibit a statistically significant difference. The immediate TAD (TAD_postOP_) post-operation was 23.97 and 25.22 for the GS and Affixus groups, respectively, but this difference was not statistically significant. In terms of the Cleveland index, the GS group had 1 case in zone 2, 29 cases in zone 5, 1 case in zone 6, 8 cases in zone 8, and 4 cases in zone 9. In the Affixus group, there was 1 case in zone 1, 1 case in zone 2, 4 cases in zone 4, 26 cases in zone 5, 3 cases in zone 6, 10 cases in zone 8, and 1 case in zone 9. However, this difference was also not statistically significant. The NSA on the contralateral side measured 123.06 for the GS group, which was statistically and significantly smaller compared to 132.25 for the Affixus group. However, the NSA on the operative side measured 130.39 and 130.82 for the GS and Affixus groups, respectively, with no statistically significant difference. As a result, the difference between these two values was 7.33 for the GS group and −1.44 for the Affixus group, indicating that the GS group achieved a greater valgus reduction (Table 4).

At 6 months post-operation, the TAD (TAD_6m_) was 23.18 for the GS group and 22.94 for the Affixus group. The difference between immediate postoperative and 6-month postoperative examinations (TAD difference = TAD_6m_ − TAD_postOP_) was −0.79 and −2.28 in the GS and Affixus groups, respectively, but this difference was not statistically significant. Regarding the NSA at 6 months (NSA_6m_), the GS group had a value of 120.31, whereas the Affixus group had 126.20, showing a statistically significant difference. The difference between the NSA both immediately post-operation and at 6 months (NSA difference = NSA_6m_ − NSA_postOP_) was −10.08 for the GS group and −4.61 for the Affixus group, indicating a statistically significant varus change in the GS group compared to the Affixus group (Table 5).

In the case of advancement distance, the GS group had a value of 1.89, which was slightly less than the Affixus group at 2.05, but this difference was not statistically significant. Conversely, the sliding distance was higher in the GS group with a value of 3.87 compared to 3.73 in the Affixus group, but again, this difference did not reach statistical significance (Table 6).

**Figure 4:**
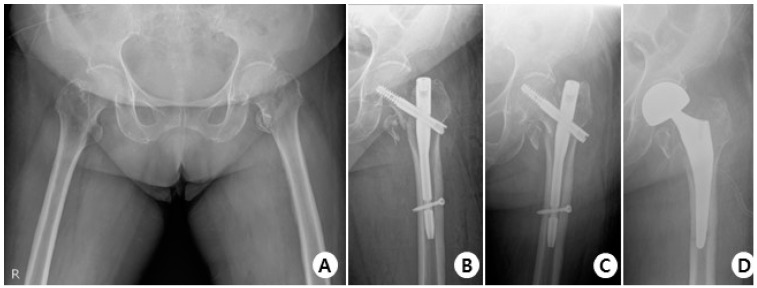
(**A**) Total hip AP X-ray of a 76-year-old female diagnosed with an intertrochanteric fracture of the left hip. (**B**) Immediately post-operation, the fractured segment was reduced in a slight valgus position using the GS hip nail implant. (**C**) The two months postoperative X-ray shows reduction loss and the progression of varus deformity, with the lag screw experiencing cut-out. (**D**) X-rays show the revision surgery performed with arthroplasty.

In terms of complications, in the GS group, there were 38 cases with no complications, 3 cases of cut-out (Figure 4), 1 case of non-union, and 1 case of delayed union.

**Figure 5:**
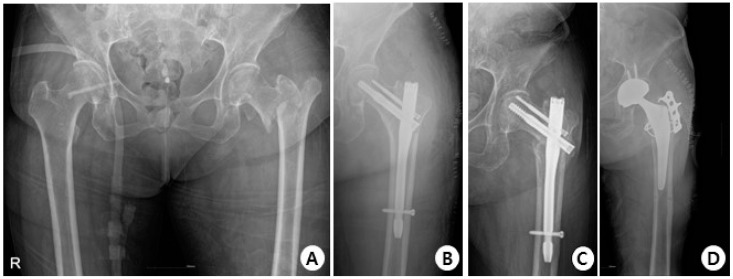
(**A**) Total hip AP X-ray of a 77-year-old female diagnosed with an intertrochanteric fracture of the left hip. (**B**) Immediately post-operation, reduction was performed using the Affixus hip fracture nail, but there appeared to be a mismatch in rotation. (**C**) The postoperative six-month X-ray shows reduction loss and the progression of varus deformity, with the lag screw experiencing cut-out. (**D**) X-rays show the revision surgery performed with arthroplasty.

In the Affixus group, there were 34 cases with no complications, 5 cases of cut-out (Figure 5), 1 case of cut through, 4 cases of non-union, 1 case of delayed union, and 1 case of osteolysis. 

There was no statistically significant difference in the occurrence of complications between the two groups, and neither group experienced implant breakage (Table 7).

### 3.4. Clinical Outcomes

In terms of clinical outcomes, the GS group had an mHHS of 50.00, while the Affixus group had a higher mHHS of 54.98. In terms of EQ-5D-5L, the GS group had a score of 0.47, while the Affixus group had a higher score of 0.60. However, there was no statistically significant difference between these two groups for both mHHS and EQ-5D-5L scores (Table 8).

### 3.5. Financial Outcomes

In terms of financial outcomes, the GS group had a higher implant cost of USD 611.78 compared to USD 590.53 in the Affixus group. However, the total hospital cost for the GS group was lower at USD 2471.92 compared to USD 2529.12 in the Affixus group, and these figures showed no statistically significant difference (Table 9).

## 4. Discussion

Intertrochanteric fractures have their limitations in conservative treatment, and surgical intervention is the primary and often the only effective treatment method. While there has been considerable debate in the past, there has been a growing trend toward surgical treatment using intramedullary nailing in recent years [8]. This is because early mobilization through surgical fixation is highly important, and closed reduction does not compromise the biology of the fracture site, including essential elements like hematoma, which are crucial for bone healing [30,31]. Furthermore, it minimizes soft tissue dissection, leading to reduced blood loss and a decreased risk of infection, wound complications, and trauma damage [32,33]. However, there is still difficulty in choosing which type of nail implant to use for surgery due to the numerous options available to surgeons [34,35,36]. In particular, Asians tend to have a shorter overall height compared to Western populations such as Europeans or Americans, resulting in a smaller femoral length and diameter [37]. For this reason, when using existing implants developed in the United States or Europe, fixation loosening has been observed in individual patients due to variations in proximal femoral geometry and the compatibility of the implant [38,39].

In this study, there were no statistically significant differences between the two groups in demographic data, excluding hospital stay. These demographic factors included age, gender, BMI, BMD, time to operation, preoperative Hb level, smoking status, ASA score, Koval grade, and fracture classification. In a study conducted by Lefaivre et al. [40], it was emphasized that early surgical intervention is the most effective approach to reduce mortality and postoperative complications following a fracture. Furthermore, Leung et al. [41] stated that surgical treatment within 48 h of fracture occurrence resulted in a lower incidence of complications such as pulmonary embolism, DVT, stroke, arrhythmias, pneumonia, and sepsis compared to surgical treatment beyond 48 h. They also mentioned that the incidence of these complications increases exponentially with each day that passes from the traumatic episode. While there was no statistically significant difference, it is worth considering that the time to operation after the occurrence of the fracture was longer for the GS group, with 3.93 days compared to 2.46 days in the Affixus group. This is an aspect to take into account when interpreting the data.

The difference in postoperative Hb levels measured on the first day postoperatively and their respective preoperative Hb values did not show a statistically significant difference between the two groups. However, the operation time was significantly shorter in the GS group, with a mean of 43.26 min compared to 51.11 min in the Affixus group. It is noteworthy that the surgery was performed by a single surgeon, and even considering the insertion of the anti-rotation screw with the Affixus hip fracture nail, the GS group’s operation time was approximately 8 min shorter, which is a significant finding.

In the postoperative radiologic evaluation, there was no significant difference between the two groups in terms of reduction quality, the TAD, and the Cleveland index. However, it was observed that the contralateral side’s NSA in the GS group had a significantly greater varus angle. Considering that the operative side’s NSA showed no significant difference between the groups immediately after the operation, it suggests that the GS group underwent a reduction at a significantly greater valgus angle compared to the native anatomy. This became more evident in the 6-month follow-up radiologic evaluation, where a pronounced difference was observed. The GS group, which had a greater valgus reduction, actually experienced a varus collapse of approximately 10 degrees, from 130.39 degrees to 120.31 degrees, after 6 months. By contrast, the Affixus group showed a lower varus collapse of about 4 degrees, decreasing from 130.82 degrees to 126.20 degrees during the same period. Many previous studies have reported that if varus collapse occurs postoperatively, it eventually leads to one of the most common and critical mechanical complications of intramedullary nailing, known as cut-out [42,43,44,45,46]. Furthermore, Durusoy et al. [47] reported that intramedullary nail movement occurs depending on the diameter of the nail and the fracture type, and as the movement increases, varus collapse progresses. However, in this study, it was reported that there was no statistically significant difference in the occurrence rate of complications such as cut-outs between the two groups, and the proportion of cut-out occurrence in the GS group itself was reported to be small. This suggests that actual patients are not progressing toward varus collapse but rather returning to their original native anatomy. In fact, the difference between the contralateral side and the NSA at 6 months indicated that the GS group actually progressed less in their varus angle.

At the 6-month follow-up radiologic evaluation, there was no significant difference between the two groups in terms of advancement distance and sliding distance. According to Shin et al. [48], a lag screw migration of more than 10mm can lead to subcutaneous irritation and bursitis, but in both groups, the migration was minimal at 3.87 and 3.73, respectively, which falls within an acceptable range. From a complication perspective, Marbrouk et al. [18] reported an overall complication rate requiring further surgery of 3%. The rate of cut-out occurrence was 1%, back-out incidence was also 1%, and fracture healing was achieved in all patients. Additionally, there were no reported cases of implant breakage. In other studies, the overall mortality rate was 6.3%, with non-union and cut-out rates at 2.7%, while the need for revision surgery occurred in 2.5% of cases [49]. In this study, the cut-out occurrence rate was 7% in the GS group and 10.9% in the Affixus group. The non-union incidence rate was also relatively high, with 2.3% in the GS group and 8.7% in the Affixus group. However, it was reported that there was no statistically significant difference between these two groups.

Although there were slight differences favoring the Affixus group in clinical outcomes, as mentioned earlier, this can likely be attributed to the longer time to operation in the GS group. However, it is important to note that these differences did not reach statistical significance, so they may not be considered noteworthy results. In terms of implant cost, it was observed that the GS group incurred slightly higher costs compared to the Affixus group. On the other hand, from the perspective of the total hospital cost for patients, the GS group was found to be less expensive. This is likely due to the shorter hospital stay in the GS group, as mentioned earlier, and since no statistically significant differences were observed, these findings may not be considered particularly significant.

This study has several limitations. First, it is a retrospective study, not a prospective one. While demographic data did not statistically differ between the two groups, patient matching was not performed. Additionally, as this study focused on fracture patients, native femoral NSA had to be inferred from the NSA of the contralateral side, introducing potential bias due to asymmetry between both femurs. It should be noted that this study only provides short-term outcomes, and long-term follow-up is necessary in the future. Furthermore, the sample size of patients was relatively small, warranting larger-scale research. However, despite these limitations, this study holds value as it is the first unique research conducted on the GS hip nail after its introduction into clinical practice. Moreover, it benefits from being a single-center study performed by a single surgeon, which adds strength to its findings.

## 5. Conclusions

This study serves as an initial exploration into the clinical outcomes of the GS hip nail, highlighting its unique attributes. Notably, it demonstrates that the GS hip nail procedure, performed by a single surgeon, significantly reduces operation time compared to the Affixus hip fracture nail, indicating its efficiency. Moreover, both radiologic and clinical outcomes appear to be comparable when compared with the Affixus hip fracture nail. However, it is important to recognize that this study provides short-term outcomes, and future research should include long-term follow-up to fully evaluate the performance of the GS hip nail. Additionally, a larger patient study should be considered for a more comprehensive understanding of its effectiveness. This research lays the foundation for further investigations into the GS hip nail’s role in improving patient outcomes in the treatment of hip fractures.

## Figures and Tables

**Table 1:** Demographic data.

Factors	GS Hip Nail	Affixus	*p*-Value
	(N = 43)	(N = 46)	
	n (%)	n (%)	
**Age (years)**			1.0000 ^§§^
mean ± SD	73.02 ± 11.49	73.20 ± 10.54	
**Gender**			0.3116 ^†^
Male	16 (37.21)	22 (47.83)	
Female	27 (62.79)	24 (52.17)	
**BMI (kg/m^2^)**			0.1221 ^§^
mean ± SD	22.73 ± 3.69	23.97 ± 3.53	
**BMD**			0.1974 ^§^
mean ± SD	−3.03 ± 1.10	−2.73 ± 1.11	
**Time to OP (days)**			0.0717 ^§§^
mean ± SD	3.93 ± 5.45	2.46 ± 2.30	
**Hospital stay (days)**			0.0002 ^§§^
mean ± SD	13.42 ± 5.46	17.80 ± 5.92	
**Preoperative Hb**			0.9367 ^§^
mean ± SD	12.08 ± 1.77	12.05 ± 1.68	

n = Number of subjects, % = Proportion of subjects, SD = Standard Deviation. BMI = Body mass index, BMD = Body mass density, OP = Operation, Hb = Hemoglobin. ^†^: Pearson’s chi-square test. ^§^: Two-sample *t*-test. ^§§^: Wilcoxon’s rank sum test.

**Table 2:** Clinical characteristics.

Factors	GS Hip Nail	Affixus	*p*-Value
	(N = 43)	(N = 46)	
	n (%)	n (%)	
**Smoking status**			0.5132 ^‡^
Yes	6 (13.95)	4 (8.70)	
No	37 (86.05)	42 (91.30)	
**ASA score**			1.0000 ^‡^
1	0 (0.00)	0 (0.00)	
2	26 (60.47)	28 (60.87)	
3	17 (39.53)	18 (36.96)	
**Koval grade**			0.2870 ^‡^
1	28 (65.12)	28 (60.87)	
2	2 (4.65)	7 (15.22)	
3	3 (6.98)	1 (2.17)	
4	5 (11.63)	8 (17.39)	
5	3 (6.98)	2 (4.35)	
6	2 (4.65)	0 (0.00)	
7	0 (0.00)	0 (0.00)	
**AO/OTA**			0.8274 ^‡^
11	10 (23.26)	8 (17.39)	
12	8 (18.60)	5 (10.87)	
13	1 (2.33)	2 (4.35)	
21	7 (16.28)	10 (21.74)	
22	10 (23.26)	10 (21.74)	
23	2 (4.65)	6 (13.04)	
31	2 (4.65)	2 (4.35)	
32	0 (0.00)	0 (0.00)	
33	3 (6.98)	3 (6.52)	

n = Number of subjects, % = Proportion of subjects. AO/OTA = Arbeitsgemeinschaft für Osteosynthesefragen/Orthopaedic Trauma Association. ASA = American Society of Anesthesiologists. ^‡^: Fisher’s exact test.

**Table 3:** Intraoperative evaluation.

Factors	GS Hip Nail	Affixus	*p*-Value
	(N = 43)	(N = 46)	
**OP time (hours)**			0.0001 ^§^
mean ± SD	43.26 ± 9.38	51.11 ± 9.15	
**Postoperative Hb**			0.7440 ^§^
mean ± SD	9.26 ± 1.31	9.16 ± 1.59	
**Difference ^1)^**			0.7457 ^§§^
mean ± SD	−2.82 ± 1.37	−2.89 ± 1.31	

n = Number of subjects, % = Proportion of subjects, SD = Standard Deviation. OP = Operation, Hb = Hemoglobin. ^§^: Two-sample *t*-test. ^§§^: Wilcoxon’s rank sum test. ^1)^ Difference = Postoperative Hb-Preoperative Hb.

**Table 4:** Radiologic evaluation (postoperative).

Factors	GS Hip Nail	Affixus	*p*-Value
	(N = 43)	(N = 46)	
	n (%)	n (%)	
**Reduction**			0.1814 ^†^
Good	17 (39.53)	19 (41.30)	
Acceptable	14 (32.56)	21 (45.65)	
mean ± SD	12 (27.91)	6 (13.04)	
**TAD_postOP_ (mm)**			0.5794 ^§§^
mean ± SD	23.97 ± 8.23	25.22 ± 8.69	
**Cleveland index**			0.2098 ^‡^
1	0 (0.00)	1 (2.17)	
2	1 (2.33)	1 (2.17)	
3	0 (0.00)	0 (0.00)	
4	0 (0.00)	4 (8.70)	
5	29 (67.44)	26 (56.52)	
6	1 (2.33)	3 (6.54)	
7	0 (0.00)	0 (0.00)	
8	8 (18.60)	10 (21.74)	
9	4 (9.30)	1 (2.17)	
**Neck-shaft angle (NSA)_postOP_**			
**Contralateral side**			<0.0001 ^§^
mean ± SD	123.06 ± 5.28	132.25 ± 7.09	
**Operative side**			0.7831 ^§^
mean ± SD	130.39 ± 7.98	130.82 ± 6.48	
**Difference ^1)^**			<0.0001 ^§^
mean ± SD	7.33 ± 9.23	−1.44 ± 9.17	

n = Number of subjects, % = Proportion of subjects, SD = Standard Deviation. TAD = Tip apex distance, NSA = Neck-shaft angle. ^†^: Pearson’s chi-square test. ^‡^: Fisher’s exact test. ^§^: Two-sample *t*-test. ^§§^: Wilcoxon’s rank sum test. ^1)^ Difference = Operative side–Contralateral side.

**Table 5:** Radiologic evaluation (follow up 6 months).

Factors	GS Hip Nail	Affixus	*p*-Value
**TAD_6m_**			0.8895 ^§^
n	41	45	
mean ± SD	23.18 ± 7.22	22.94 ± 8.52	
**TAD difference ^1)^**			0.7100 ^§§^
n	41	45	
mean ± SD	−0.79 ± 5.45	−2.28 ± 6.27	
**NSA_6m_**			<0.0001 ^§^
n	43	46	
mean ± SD	120.31 ± 7.47	126.20 ± 5.78	
**NSA difference ^2)^**			0.0002 ^§§^
n	43	46	
mean ± SD	−10.08 ± 8.16	−4.61 ± 5.48	

n = Number of subjects, SD = Standard Deviation. TAD = Tip apex distance, NSA = Neck-shaft angle. ^§^: Two-sample *t*-test. ^§§^: Wilcoxon’s rank sum test. ^1)^ Difference = TAD_6m_–TAD_postOP._^2)^ Difference = NSA_6m_–NSA_postOP._

**Table 6:** Radiologic distance (follow up 6 months).

Factors	GS Hip Nail	Affixus	*p*-Value
	(N = 43)	(N = 46)	
**Advancement distance**			0.8023 ^§§^
mean ± SD	1.89 ± 3.36	2.05 ± 5.02	
**Sliding distacne**			0.7896 ^§§^
mean ± SD	3.87 ± 2.56	3.73 ± 4.96	

n = Number of subjects, SD = Standard Deviation. ^§§^: Wilcoxon’s rank sum test.

**Table 7:** Radiologic evaluation (complication).

Factors	GS Hip Nail	Affixus	*p*-Value
	(N = 43)	(N = 46)	
	n (%)	n (%)	
none	38 (88.37)	34 (73.91)	0.4956 ^‡^
cut out	3 (6.98)	5 (10.87)	
cut through	0 (0.00)	1 (2.17)	
back out	0 (0.00)	0 (0.00)	
non-union	1 (2.33)	4 (8.70)	
delayed union	1 (2.33)	1 (2.17)	
osteolysis	0 (0.00)	1 (2.17)	
implant-breakage	0 (0.00)	0 (0.00)	

n = Number of subjects, SD = Standard Deviation. ^‡^: Fisher’s exact test.

**Table 8:** Clinical Outcomes.

Factors	GS Hip Nail	Affixus	*p*-Value
**mHHS**			0.1766 ^§§^
n	43	41	
mean ± SD	50.00 ± 23.67	54.98 ± 32.43	
**EQ-5D-5L score**			0.0792 ^§§^
n	43	46	
mean ± SD	0.47 ± 0.32	0.60 ± 0.35	

n = Number of subjects, SD = Standard Deviation. mHHS = modified Harris hip score, EQ-5D-5L = European Quality of Life-5 Dimensions-5 Levels. §§: Wilcoxon’s rank sum test.

**Table 9:** Financial Outcomes.

Factors	GS Hip Nail	Affixus	*p*-Value
**Cost [$]**			
**Implant cost**			0.0936 ^§§^
n	43	46	
mean ± SD	611.78 ± 64.38	590.53 ± 47.26	
**Hospital cost**			0.5104 ^§§^
n	40	44	
mean ± SD	2471.92 ± 1071.02	2529.12 ± 1208.01	

n = Number of subjects, SD = Standard Deviation. ^§§^: Wilcoxon’s rank sum test.

## Data Availability

All data generated during this study are included in this published article.
